# Efficacy and safety of canagliflozin monotherapy in subjects with type 2 diabetes mellitus inadequately controlled with diet and exercise

**DOI:** 10.1111/dom.12054

**Published:** 2013-01-24

**Authors:** K Stenlöf, W T Cefalu, K-A Kim, M Alba, K Usiskin, C Tong, W Canovatchel, G Meininger

**Affiliations:** 1 Clinical Trial Center, Sahlgrenska University HospitalGothenburg, Sweden; 2 Pennington Biomedical Research CenterBaton Rouge, LA, USA; 3 LSUHSC School of MedicineNew Orleans, LA, USA; 4 Department of Internal Medicine, Dongguk University Ilsan Hospital, Dongguk University School of MedicineGoyang, South Korea; 5 Janssen Research & Development, LLCRaritan, NJ, USA

**Keywords:** phase 3 study, SGLT2 inhibitor, type 2 diabetes

## Abstract

**Aims:**

Canagliflozin is a sodium glucose co-transporter 2 inhibitor in development for type 2 diabetes mellitus (T2DM). The efficacy and safety of canagliflozin were evaluated in subjects with T2DM inadequately controlled with diet and exercise.

**Methods:**

In this 26-week, randomized, double-blind, placebo-controlled, phase 3 trial, subjects (N = 584) received canagliflozin 100 or 300 mg or placebo once daily. Primary endpoint was the change from baseline in haemoglobin A1c (HbA1c) at week 26. Secondary endpoints included the proportion of subjects achieving HbA1c < 7.0%; change from baseline in fasting plasma glucose (FPG), 2-h postprandial glucose (PPG) and systolic blood pressure (BP); and percent change in body weight, high-density lipoprotein cholesterol (HDL-C) and triglycerides. Adverse events (AEs) were recorded throughout the study.

**Results:**

At week 26, HbA1c was significantly reduced from baseline with canagliflozin 100 and 300 mg compared with placebo (−0.77, −1.03 and 0.14%, respectively; p < 0.001 for both). Both canagliflozin doses significantly decreased FPG, 2-h PPG, body weight and systolic BP (p < 0.001 for all), and increased HDL-C compared with placebo (p < 0.01 for both). Overall incidences of AEs were modestly higher with canagliflozin versus placebo; rates of serious AEs and AE-related discontinuations were low and similar across groups. Incidences of genital mycotic infections, urinary tract infections and osmotic diuresis-related AEs were higher with canagliflozin; these led to few discontinuations. The incidence of hypoglycaemia was low across groups.

**Conclusion:**

Canagliflozin treatment improved glycaemic control, reduced body weight and was generally well tolerated in subjects with T2DM inadequately controlled with diet and exercise.

## Introduction

The substantial increase in the incidence of type 2 diabetes mellitus (T2DM) over the past decade is associated with a marked increase in the prevalence of obesity, which contributes greatly to insulin resistance, a key pathophysiologic parameter observed especially in individuals at risk. Given the role of obesity and sedentary lifestyles in contributing to the progression of T2DM, the first step in T2DM management is lifestyle change, exercise and weight loss; unfortunately, such interventions are often inadequate or short-lived in effectiveness. When non-pharmacological treatment fails, treatment with an antihyperglycaemic agent (AHA), often metformin, is initiated [1]. Metformin provides effective control, although some patients do not tolerate metformin due to gastrointestinal side-effects or have contraindications to the use of this agent, such as renal insufficiency. Type 2 diabetes is a progressive disease; initially, inadequate β-cell compensation for the increased demand in insulin due to insulin resistance precipitates hyperglycaemia; subsequent deterioration in β-cell function (βCF) and mass underlies the progression from non-pharmacological treatment through monotherapy failure, to the need for combination treatments [1–4]. Current oral AHA classes often do not provide sufficiently effective or durable glycaemic control with improved βCF; although metformin provides modest weight reduction, most oral agents lead to weight gain or are weight neutral, but do not substantively reduce body weight [1,5]. The recent American Diabetes Association/European Association for the Study of Diabetes position statement suggests that therapy should be individualized and tailored to the specific needs of each patient [1]. Therefore, there is a need for new AHAs that can provide long-term glycaemic control and additional benefits such as minimal hypoglycaemia and favourable effects on weight.

Sodium glucose co-transporter 2 (SGLT2) inhibitors are a class of AHAs in development that have a mechanism of action different from those of current therapies, with a primary effect on renal glucose handling. Specifically, induction of urinary glucose excretion (UGE) via inhibition of renal glucose reabsorption by SGLT2 provides an insulin-independent mechanism for lowering blood glucose and improving glycaemic control [6]. Under normal conditions, almost all filtered glucose is reabsorbed until the filtered load exceeds the glucose resorptive capacity. The plasma glucose concentration at which renal resorptive capacity is exceeded and UGE occurs is called the renal threshold for glucose (RT_G_). Renal glucose resorptive capacity is increased in T2DM, contributing to the worsening of hyperglycaemia [7]. Canagliflozin, an SGLT2 inhibitor in development for the treatment of T2DM, lowers the RT_G_ (range of 4.4–5.0 mmol/l), thereby increasing UGE and resulting in decreased plasma glucose, a mild osmotic diuresis and increased caloric loss (4 kcal/g of glucose), with a low potential for hypoglycaemia [8–13]. The loss of glucose with attendant caloric loss contributes to weight loss; in addition, improvements in βCF have been seen [8,11]. This clinical profile suggests that canagliflozin might be a useful therapeutic agent to treat patients with T2DM from early in the disease, as monotherapy, to later in the disease, in combination treatments. This 26-week, phase 3, CANagliflozin Treatment And Trial Analysis – Monotherapy (CANTATA-M) study evaluated the efficacy and safety of canagliflozin compared with placebo in subjects with T2DM inadequately controlled with diet and exercise.

## Materials and Methods

### Subjects and Study Design

This randomized, double-blind, placebo-controlled, phase 3 study was conducted in 17 countries (ClinicalTrials.gov identifier: NCT01081834). The study included both subjects with inadequate control on diet and exercise and subjects on an AHA, who underwent a washout of the agent. Subjects not on an AHA directly entered a 2-week, single-blind, placebo run-in period (week −2 to day 1), while subjects on an AHA underwent an 8-week, AHA washout/diet and exercise period followed by the placebo run-in period. After the placebo run-in period, all subjects were randomized into a 26-week, double-blind, placebo-controlled, core treatment period, followed by a 26-week, double-blind extension period. This publication reports the results of the 26-week core treatment period.

Eligible subjects were men and women 18–80 years of age with T2DM who met one of the two following criteria: (i) not on an AHA at screening with haemoglobin A1c (HbA1c) ≥7.0 and ≤10.0% or (ii) on AHA monotherapy [except peroxisome proliferator-activated receptor-γ (PPARγ) agonist] or metformin plus sulfonylurea combination therapy (at ≤50% of maximally or near-maximally effective doses) with HbA1c ≥6.5 and ≤9.5% at screening and HbA1c ≥7.0 and ≤10.0% and fasting plasma glucose (FPG) <15.0 mmol/l at week −2.

For subjects with HbA1c values above the inclusion range (HbA1c ≥7.0 and ≤10.0%), a substudy was conducted to assess the efficacy in elevated glycaemic states. Subjects were eligible to participate in the high glycaemic substudy if they had HbA1c >10.0 and ≤12.0% at screening or week −1 and FPG ≤19.4 mmol/l at week −1. Subjects eligible for this substudy entered a 1-week, single-blind, placebo run-in period followed by a 26-week, double-blind, active-treatment period. Given the poorer glycaemic control, all subjects received active treatment with canagliflozin 100 or 300 mg; double-blinding was to the dose of canagliflozin. Subjects in the high glycaemic substudy were not eligible for the 26-week extension period. In this report, the placebo-controlled study component will be referred to as the ‘main study’.

Subjects were excluded if they had repeated FPG measurements >15.0 mmol/l during the pre-treatment phase (or >19.4 mmol/l for the high glycaemic substudy); a history of type 1 diabetes, hereditary glucose-galactose malabsorption, primary renal glucosuria or cardiovascular (CV) disease (including myocardial infarction, unstable angina, revascularization procedure or cerebrovascular accident); treatment with a PPARγ agonist, insulin, another SGLT2 inhibitor or any other AHA except as specified in the inclusion criteria within 12 weeks before screening; or estimated glomerular filtration rate (eGFR) <50 ml/min/1.73 m^2^ at screening.

Subjects in the main study were randomly assigned to receive daily oral doses of canagliflozin 100 or 300 mg or placebo (1 : 1 : 1). Randomization was stratified according to whether subjects were taking AHAs at screening and whether they participated in the frequently-sampled mixed-meal tolerance test (FS-MMTT). Subjects in the high glycaemic substudy were randomly assigned to receive canagliflozin 100 or 300 mg (1 : 1), with randomization stratified by whether subjects were taking AHAs at screening.

During the double-blind treatment period, glycaemic rescue therapy with metformin was initiated if FPG >15.0 mmol/l after day 1 to week 6, >13.3 mmol/l after week 6 to week 12 and >11.1 mmol/l after week 12 to week 26.

The study protocol and amendments were approved by the institutional review boards at participating institutions and the study was conducted under the guidelines of Good Clinical Practices and the Declaration of Helsinki. All subjects provided written informed consent prior to participation.

### Study Endpoints and Assessments

The pre-specified primary efficacy endpoint was the change in HbA1c from baseline to week 26. Pre-specified secondary endpoints included the proportion of subjects reaching HbA1c <7.0%, changes from baseline at week 26 in FPG and systolic blood pressure (BP) and percent changes from baseline in body weight, high-density lipoprotein cholesterol (HDL-C) and triglycerides. Additional endpoints included changes in diastolic BP and other fasting plasma lipids, including low-density lipoprotein cholesterol (LDL-C), non–HDL-C and the LDL-C/HDL-C ratio. Change from baseline in apolipoprotein B (Apo B) was assessed in a subset of subjects in the main study (based on availability of paired baseline and week 26 archive samples).

On day 1 and at week 26, all subjects underwent a standard MMTT (∼700 kcal and 100 g of carbohydrates) to assess the pre-specified secondary endpoint, change from baseline in 2-h postprandial glucose (PPG), and indices of βCF, including Homeostasis Model Assessment (HOMA2-%B), proinsulin/insulin ratio and proinsulin/C-peptide ratio (*post hoc* analysis). A FS-MMTT was performed in a subset of subjects in the main study (∼50% of total subjects at selected sites) for measures of βCF including the ratio of C-peptide area under the concentration-time curve (AUC_C_) to glucose AUC (AUC_G_). During the FS-MMTT, blood samples were collected 15 min before and immediately prior to the meal, and 30, 60, 90, 120 and 180 min after the meal.

Safety and tolerability were assessed based on adverse event (AE) reports, safety laboratory tests, vital sign measurements, physical examinations and 12-lead electrocardiograms. AEs pre-specified for additional data collection included urinary tract infections (UTIs) and genital mycotic infections. Documented hypoglycaemia episodes included biochemically confirmed episodes (concurrent fingerstick or plasma glucose ≤3.9 mmol/l, irrespective of symptoms) and severe hypoglycaemia episodes (i.e., requiring the assistance of another individual or resulting in seizure or loss of consciousness).

### Statistical Analysis

Sample size determination for the main study was based on the comparison of canagliflozin with placebo in the change in HbA1c from baseline to week 26. An estimated 85 randomized subjects per group were needed to achieve at least 90% power, assuming a group difference of 0.5% and a common standard deviation (s.d.) of 1.0%. To enhance the safety database for canagliflozin, approximately 150 randomized subjects were planned for inclusion per group. Sample size determination was not required for the high glycaemic substudy because there were no comparisons pre-specified for hypothesis testing; 50–100 subjects were targeted for enrollment to provide a reasonable experience at each dose for efficacy, safety and tolerability assessments.

Efficacy and safety analyses for the main study and the high glycaemic substudy were performed separately using the modified intent-to-treat (mITT) population consisting of all randomized subjects who received ≥1 dose of the study drug. The last observation carried forward (LOCF) approach was used to impute missing efficacy data. For subjects who received rescue therapy, the last post-baseline value prior to the initiation of rescue therapy was used for the efficacy analyses.

Primary and continuous secondary endpoints were analyzed using an analysis of covariance (ANCOVA) model with treatment and stratification factors as fixed effects and the corresponding baseline value as a covariate. The least squares (LS) mean differences between groups (each canagliflozin dose versus placebo) and the associated two-sided 95% confidence intervals (CIs) were estimated based on this model. The categorical secondary efficacy endpoint (proportion of subjects reaching HbA1c <7.0%) was analyzed using a logistic model with treatment and stratification factors as fixed effects and baseline HbA1c as a covariate.

Descriptive statistics with 95% CIs were provided for the change from baseline in HbA1c for subgroups with baseline HbA1c <8%, ≥8 to <9% and ≥9%. For indices of βCF, descriptive statistics and 95% CIs for the changes from baseline were provided; LS mean differences versus placebo at week 26 were assessed using the same ANCOVA model as for the primary endpoint.

A pre-specified hierarchical testing sequence was implemented to strongly control for overall type I error due to multiplicity. Two-sided statistical tests were conducted at the 5.0% significance level for all endpoints except systolic BP, HDL-C and triglycerides. Systolic BP, HDL-C and triglycerides were grouped together into two separate families, one each for canagliflozin 100 and 300 mg. Each family was tested using the Hochberg procedure controlling for multiplicity at the 2.5% significance level. P-values were calculated by comparing LS means and are reported for pre-specified comparisons only. Descriptive statistics were provided for endpoints for the high glycaemic substudy.

All AEs are reported including all data, regardless of rescue medication, except for osmotic diuresis- and volume-related AEs, which are reported excluding data after initiation of rescue therapy. Safety analyses for laboratory results excluded data after initiation of rescue therapy, including data up to within 2 days after the last dose of study drug.

## Results

### Subject Disposition and Baseline Characteristics

#### Main Study

**Figure 1:**
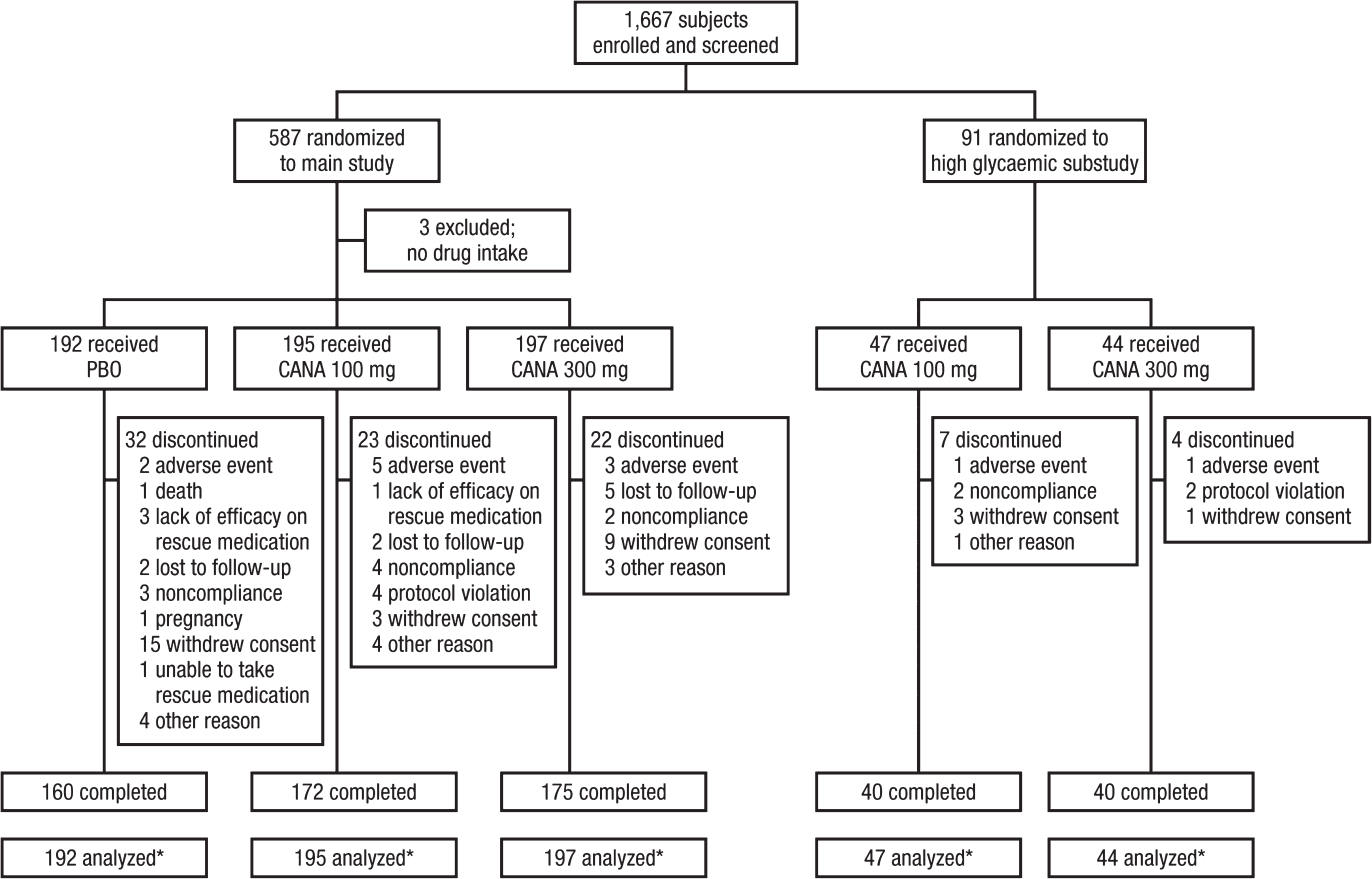
Study flow diagram. CANA, canagliflozin; mITT, modified intent-to-treat; PBO, placebo. ^*^mITT analysis set.

Among the 587 subjects randomized in the main study, 584 received ≥1 dose of study medication and were included in the mITT analysis set (figure 1). A total of 77 (13.1%) subjects discontinued prior to week 26; slightly higher rates of discontinuation were seen with placebo (16.5%) than with canagliflozin 100 and 300 mg (11.7 and 11.2%, respectively). A lower percentage of subjects treated with canagliflozin 100 and 300 mg (2.6 and 2.0%, respectively) received glycaemic rescue therapy compared with placebo-treated subjects (22.7%). Demographic and baseline characteristics were generally balanced across treatment groups (Table 1).

**Table 1:** Baseline demographics and disease characteristics*

	Main study				High glycaemic substudy		
		
Characteristic	PBO (n = 192)	CANA 100 mg (n = 195)	CANA 300 mg (n = 197)	Total (N = 584)	CANA 100 mg (n = 47)	CANA 300 mg (n = 44)	Total (N = 91)
Sex, n (%)							
Male	88 (45.8)	81 (41.5)	89 (45.2)	258 (44.2)	23 (48.9)	19 (43.2)	42 (46.2)
Female	104 (54.2)	114 (58.5)	108 (54.8)	326 (55.8)	24 (51.1)	25 (56.8)	49 (53.8)
Age (years)	55.7 (10.9)	55.1 (10.8)	55.3 (10.2)	55.4 (10.6)	49.7 (11.1)	48.8 (10.8)	49.3 (10.9)
Race, n (%)†							
White	134 (69.8)	124 (63.6)	137 (69.5)	395 (67.6)	25 (53.2)	30 (68.2)	55 (60.4)
Black or African American	9 (4.7)	18 (9.2)	14 (7.1)	41 (7.0)	3 (6.4)	1 (2.3)	4 (4.4)
Asian	29 (15.1)	27 (13.8)	29 (14.7)	85 (14.6)	11 (23.4)	7 (15.9)	18 (19.8)
Other‡	20 (10.4)	26 (13.3)	17 (8.6)	63 (10.8)	8 (17.0)	6 (13.6)	14 (15.4)
Hb A1c (%)	8.0 (1.0)	8.1 (1.0)	8.0 (1.0)	8.0 (1.0)	10.6 (0.9)	10.6 (0.9)	10.6 (0.9)
FPG (mmol/l)	9.3 (2.1)	9.6 (2.4)	9.6 (2.4)	9.5 (2.3)	13.3 (3.2)	13.6 (3.2)	13.4 (3.2)
Body weight (kg)	87.6 (19.5)	85.8 (21.4)	86.9 (20.5)	86.8 (20.4)	82.8 (22.9)	82.1 (19.0)	82.5 (21.0)
BMI (kg/m^2^)	31.8 (6.2)	31.3 (6.6)	31.7 (6.0)	31.6 (6.2)	30.4 (7.1)	30.5 (5.5)	30.5 (6.3)
Duration of diabetes (years)	4.2 (4.1)	4.5 (4.4)	4.3 (4.7)	4.3 (4.4)	4.6 (4.6)	5.2 (4.8)	4.9 (4.7)
Subjects on AHA at screening, n (%)	92 (47.9)	94 (48.2)	95 (48.2)	281 (48.1)	11 (23.4)	10 (22.7)	21 (23.1)

AHA, antihyperglycaemic agent; BMI, body mass index; CANA, canagliflozin; FPG, fasting plasma glucose; HbA1c, haemoglobin A1c; PBO, placebo; s.d., standard deviation.

* Data are mean (s.d.) unless otherwise indicated.

† Percentages may not total 100.0% due to rounding.

‡ Including American Indian or Alaska Native, other, unknown and not reported for the main study and American Indian or Alaska Native and other for the high glycaemic substudy.

#### High Glycaemic Substudy

All 91 subjects who participated in the high glycaemic substudy were included in the mITT analysis set (figure 1 and Table 1). Eleven (12.1%) subjects discontinued before week 26; rates of discontinuation were similar across both canagliflozin groups. Subjects in the high glycaemic substudy had a mean baseline HbA1c of 10.6%. In this cohort, 23.1% of subjects were on AHA therapy at screening compared with 48.1% of subjects in the main study, consistent with the higher baseline glycaemia in this population.

### Efficacy

#### Glycaemic Efficacy Endpoints

##### Main study

**Figure 2:**
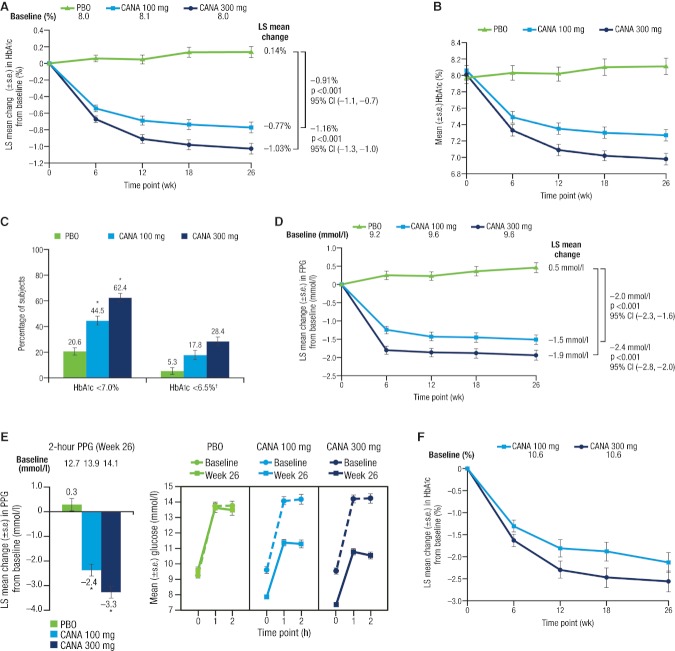
Changes in glycaemic parameters (LOCF). (A) Change in HbA1c, (B) mean HbA1c over time, (C) proportion of subjects reaching HbA1c goals, (D) change in FPG, (E) change in PPG and (F) change in HbA1c (high glycaemic substudy). CANA, canagliflozin; CI, confidence interval; FPG, fasting plasma glucose; HbA1c, haemoglobin A1c; LOCF, last observation carried forward; LS, least squares; PBO, placebo; PPG, postprandial glucose; s.e., standard error. *p < 0.001 versus PBO. ^†^Statistical comparison for CANA 100 and 300 mg versus PBO not performed (not pre-specified).

At week 26, canagliflozin 100 and 300 mg provided significant reductions in HbA1c from baseline compared with placebo (p < 0.001 for both canagliflozin doses; figures 2A and 2B). Differences in LS mean changes were −0.91 and −1.16% with canagliflozin 100 and 300 mg relative to placebo, respectively. In both canagliflozin groups, a substantial reduction from baseline in HbA1c was observed by week 12, with modest progressive reductions and no apparent plateau observed through week 26. The decrease in HbA1c was similar between subjects who were not on an AHA at screening (52%) and those who underwent AHA washout. Compared with placebo, a greater proportion of subjects achieved HbA1c <7.0% (p < 0.001 for both canagliflozin doses) and HbA1c <6.5% at week 26 with canagliflozin 100 and 300 mg (figure 2C). Subgroup analyses based on baseline HbA1c (<8%, ≥8 to <9% and ≥9%) showed that HbA1c reductions with canagliflozin were greater in the subgroup with higher baseline HbA1c; however, sizeable reductions were also seen in the subgroup with the lowest baseline HbA1c (figure S1).

Canagliflozin 100 and 300 mg provided significantly greater reductions in FPG over 26 weeks compared with placebo (figure 2D). Reductions in FPG in the canagliflozin groups were near maximal by week 6, with a slight progressive decline through week 26, and with a modest rise in FPG from baseline in the placebo group. At week 26, differences in LS mean changes in FPG were −2.0 and −2.4 mmol/l for canagliflozin 100 and 300 mg relative to placebo, respectively (p < 0.001 for both canagliflozin doses). Substantial reductions in 1- and 2-h PPG after a standard MMTT were observed with canagliflozin 100 and 300 mg; differences in LS mean changes for 2-h PPG were −2.7 and −3.6 mmol/l, respectively (figure 2E; p < 0.001 for both canagliflozin doses).

##### High glycaemic substudy

In subjects in the high glycaemic substudy, canagliflozin 100 and 300 mg provided reductions from baseline in HbA1c of −2.13 and −2.56%, respectively, at week 26 (figure 2F). Both canagliflozin doses were associated with large reductions from baseline in FPG and 2-h PPG (Table S1).

#### Body Weight, BP and Lipids

##### Main study

**Figure 3:**
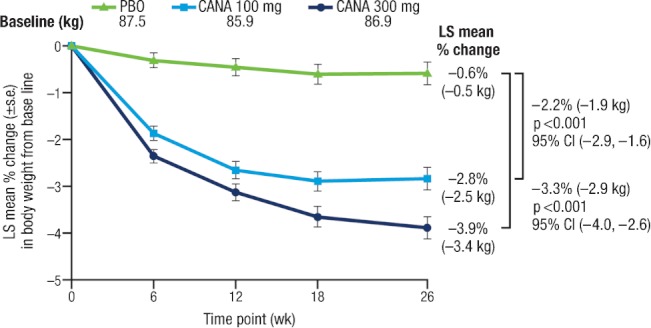
Percent change in body weight (LOCF). CANA, canagliflozin; CI, confidence interval; LOCF, last observation carried forward; LS, least squares; PBO, placebo; s.e., standard error.

Significant dose-related reductions from baseline in body weight were observed at week 26 with canagliflozin 100 and 300 mg compared with placebo (p < 0.001 for both canagliflozin doses; figure 3). Canagliflozin 100 and 300 mg provided LS mean percent changes of −2.2% (−1.9 kg) and −3.3% (−2.9 kg), respectively, relative to placebo. Weight loss with canagliflozin 100 and 300 mg occurred rapidly through week 6; a progressive decrease was seen with canagliflozin 300 mg, whereas canagliflozin 100 mg showed smaller reductions over the remaining treatment period. A small decrease in body weight was observed with placebo through week 18.

Canagliflozin 100 and 300 mg were associated with statistically significant reductions from baseline in systolic BP at week 26 compared with placebo (difference in LS mean changes versus placebo of −3.7 and −5.4 mmHg, respectively; p < 0.001 for both canagliflozin doses; Table 2). Diastolic BP was also reduced with both canagliflozin doses compared with placebo [difference in LS mean changes versus placebo of −1.6 and −2.0 mmHg, respectively; statistical comparison not performed (not pre-specified)]. Minimal changes in pulse rate were observed with canagliflozin 100 and 300 mg compared with placebo (−1.6, −0.5 and 1.4 beats per min, respectively).

**Table 2:** Summary of changes from baseline in blood pressure (BP) and fasting plasma lipids at week 26 LOCF (main study)

	PBO	CANA 100 mg	CANA 300 mg
Systolic BP, n	190	192	195
Mean (s.d.) baseline (mmHg)	127.7 (13.7)	126.7 (12.5)	128.5 (12.7)
LS mean (s.e.) change	0.4 (0.8)	−3.3 (0.8)	−5.0 (0.8)
Difference versus PBO (95% CI)		−3.7 (−5.9, −1.6)*	−5.4 (−7.6, −3.3)*
Diastolic BP, n	190	192	195
Mean (s.d.) baseline (mmHg)	77.4 (8.4)	77.7 (6.8)	79.1 (8.3)
LS mean (s.e.) change	−0.1 (0.5)	−1.7 (0.5)	−2.1 (0.5)
Difference versus PBO (95% CI)		−1.6 (−2.9, −0.2)†	−2.0 (−3.4, −0.7)†
Triglycerides, n	171	183	183
Mean (s.d.) baseline (mmol/l)	2.2 (1.2)	2.0 (1.2)	2.0 (1.1)
LS mean (s.e.) change	0.07 (0.07)	−0.16 (0.07)	−0.18 (0.07)
Median (IQR) percent change	0.0 (−16.4, 19.3)	−7.6 (−25.9, 19.5)	−9.7 (−27.8, 17.5)
LS mean (s.e.) percent change	7.9 (3.5)	2.5 (3.4)	−2.3 (3.4)
Difference versus PBO (95% CI)		−5.4 (−14.9, 4.1)‡	−10.2 (−19.6, −0.7)‡
LDL-C, n	169	180	181
Mean (s.d.) baseline (mmol/l)	3.1 (1.1)	3.1 (1.0)	2.9 (0.9)
LS mean (s.e.) change	−0.07 (0.05)	0.00 (0.05)	0.12 (0.05)
Median (IQR) percent change	−2.4 (−16.5, 12.2)	0.4 (−14.4, 13.9)	3.1 (−7.4, 19.5)
LS mean (s.e.) percent change	1.0 (1.9)	2.9 (1.8)	7.1 (1.8)
Difference versus PBO (95% CI)		2.0 (−3.2, 7.1)†	6.1 (0.9, 11.3)†
HDL-C, n	170	182	183
Mean (s.d.) baseline (mmol/l)	1.1 (0.3)	1.2 (0.3)	1.2 (0.3)
LS mean (s.e.) change	0.04 (0.02)	0.11 (0.02)	0.11 (0.02)
Median (IQR) percent change	3.2 (−6.6, 15.8)	9.2 (−2.3, 19.8)	8.9 (−1.0, 20.3)
LS mean (s.e.) percent change	4.5 (1.4)	11.2 (1.4)	10.6 (1.4)
Difference versus PBO (95% CI)		6.8 (2.9, 10.6)*	6.1 (2.3, 9.9)§
LDL-C/HDL-C, n	169	180	181
Mean (s.d.) baseline (mol/mol)	2.9 (1.3)	2.7 (1.0)	2.6 (0.9)
LS mean (s.e.) change	−0.16 (0.05)	−0.22 (0.05)	−0.12 (0.05)
Median (IQR) percent change	−6.3 (−19.7, 11.5)	−6.7 (−21.3, 7.3)	−2.9 (−18.3, 14.1)
LS mean (s.e.) percent change	−1.9 (1.9)	−5.8 (1.8)	−1.0 (1.8)
Difference versus PBO (95% CI)		−4.0 (−9.1, 1.2)†	0.9 (−4.3, 6.1)†
Non–HDL-C, n	170	181	180
Mean (s.d.) baseline, mmol/l	4.1 (1.2)	3.9 (1.0)	3.7 (1.0)
LS mean (s.e.) change	−0.05 (0.06)	−0.05 (0.05)	0.04 (0.05)
Median (IQR) percent change	−1.0 (−12.2, 9.2)	−0.6 (−12.0, 10.4)	−1.1 (−8.8, 13.5)
LS mean (s.e.) percent change	0.7 (1.5)	0.7 (1.5)	2.7 (1.5)
Difference versus PBO (95% CI)		−0.1 (−4.2, 4.1)†	1.9 (−2.3, 6.1)†

CANA, canagliflozin; CI, confidence interval; HDL-C, high-density lipoprotein cholesterol; IQR, interquartile range; LDL-C, low-density lipoprotein cholesterol; LOCF, last observation carried forward; LS, least squares; NS, not significant; PBO, placebo; s.d., standard deviation; s.e., standard error.

* p < 0.001 versus PBO.

† Statistical comparison versus PBO not performed (not pre-specified).

‡ p = NS versus PBO.

§ p < 0.01 versus PBO.

Significant increases in HDL-C were observed with canagliflozin 100 and 300 mg compared with placebo at week 26 [differences in LS mean changes of 6.8% (p < 0.001) and 6.1% (p < 0.01), respectively; Table 2]. Both canagliflozin doses were associated with reductions in triglycerides compared with placebo, but these differences did not reach statistical significance. Modest, dose-related increases from baseline in LDL-C were seen with canagliflozin 100 and 300 mg (2.9 and 7.1%, respectively) compared with placebo (1.0%). Small increases in non–HDL-C were observed in all groups (canagliflozin 100 and 300 mg and placebo: 0.7, 2.7 and 0.7%, respectively). In the subset of subjects who had adequate archived samples (at baseline and week 26) for analysis of Apo B (n = 349), increases of 1.2 and 3.5% with canagliflozin 100 and 300 mg, respectively, and 0.9% with placebo were seen; these changes from baseline in Apo B were similar to those observed in non–HDL-C. Consistent with a greater increase in HDL-C than in LDL-C, the LDL-C/HDL-C ratio was slightly decreased across groups.

##### High glycaemic substudy

In the high glycaemic substudy, both canagliflozin doses were associated with reductions in body weight and systolic BP that were generally similar to the changes observed with canagliflozin in the main study (Table S1). Dose-related increases in HDL-C were seen with both canagliflozin doses, as well as a modest reduction in triglycerides and a small increase in LDL-C with canagliflozin 300 mg.

#### β-cell Function (Main Study)

At week 26, improvements in βCF were observed with canagliflozin 100 and 300 mg compared with placebo (Table 3). Increases in HOMA2-%B, a measure of fasting insulin secretion, were observed with canagliflozin 100 and 300 mg compared with placebo (differences in LS mean changes of 12.4 and 22.8, respectively). Dose-related decreases in the proinsulin/insulin ratio were observed with canagliflozin 100 and 300 mg compared with placebo (differences in LS mean changes of −0.5 and −0.8 pmol/mIU, respectively); decreases in the proinsulin/C-peptide ratio were also seen with both canagliflozin doses compared with placebo (difference in LS mean changes of −0.01 nmol/nmol for both). Dose-related increases in the AUC_C_/AUC_G_ ratio were seen with canagliflozin 100 and 300 mg compared with placebo in the FS-MMTT subset (difference in LS mean changes of 41.0 and 50.7 pmol/mmol, respectively), suggesting an increase in insulin secretion relative to glucose with canagliflozin.

**Table 3:** Summary of changes in indices of β-cell function from baseline to week 26 (main study)*

	PBO	CANA 100 mg	CANA 300 mg
HOMA2-%B, n	116	133	130
Mean (s.d.) baseline	59.1 (29.7)	50.2 (31.5)	53.5 (29.9)
LS mean (s.e.) change	−2.5 (2.1)	9.9 (2.0)	20.3 (2.0)
Difference versus PBO (95% CI)		12.4 (6.6, 18.1)	22.8 (17.0, 28.6)
Proinsulin/insulin ratio, n	92	107	92
Mean (s.d.) baseline (pmol/mIU)	3.8 (2.3)	3.8 (1.6)	4.5 (4.1)
LS mean (s.e.) change	0.5 (0.2)	0.0 (0.2)	−0.3 (0.2)
Difference versus PBO (95% CI)		−0.5 (−1.1, 0.2)	−0.8 (−1.4, –0.1)
Proinsulin/C-peptide ratio, n	111	120	106
Mean (s.d.) baseline (nmol/nmol)	0.04 (0.02)	0.05 (0.02)	0.05 (0.02)
LS mean (s.e.) change	0.007 (0.00)	−0.003 (0.00)	−0.003 (0.00)
Difference versus PBO (95% CI)		−0.01 (−0.02, 0.00)	−0.01 (−0.02, 0.00)
AUC_C_/AUC_G_ ratio, n†	35	52	48
Mean (s.d.) baseline (pmol/mmol)	200.6 (97.6)	160.6 (92.7)	165.3 (66.5)
LS mean (s.e.) change	−18.8 (26.7)	22.2 (25.4)	31.9 (26.3)
Difference versus PBO (95% CI)		41.0 (19.0, 63.0)	50.7 (28.5, 72.9)

AUC_C_, C-peptide area under the curve; AUC_G_, glucose area under the curve; CANA, canagliflozin; CI, confidence interval; FS-MMTT, frequently-sampled mixed-meal tolerance test; HOMA, Homeostasis Model Assessment; LS, least squares; PBO, placebo; s.d., standard deviation; s.e., standard error.

* Statistical comparison for CANA 100 and 300 mg versus PBO not performed (not pre-specified).

† Assessed only for subjects who participated in the FS-MMTT.

### Safety

#### Main Study

The overall incidence of AEs was modestly higher for subjects treated with canagliflozin compared with placebo (Table 4). The incidence of serious AEs was low across the treatment groups. A total of 10 subjects in the canagliflozin groups (2.6%) discontinued treatment due to AEs, compared with two subjects (1.0%) in the placebo group (Table 4); no single AE term accounted for more than a single discontinuation. Two deaths occurred during the treatment period (one with placebo and one with canagliflozin 100 mg); neither was considered by the investigator to be drug-related.

**Table 4:** Summary of overall safety and selected adverse events (main study)*

	Subjects, n (%)		
	
	PBO (n = 192)	CANA 100 mg (n = 195)	CANA 300 mg (n = 197)
Any AE	101 (52.6)	119 (61.0)	118 (59.9)
AEs leading to discontinuation	2 (1.0)	6 (3.1)	4 (2.0)
AEs related to study drug†	18 (9.4)	34 (17.4)	50 (25.4)
Serious AEs	4 (2.1)	8 (4.1)	2 (1.0)
Deaths‡	1 (0.5)	1 (0.5)	0
Selected AEs			
UTI	8 (4.2)	14 (7.2)	10 (5.1)
Genital mycotic infection			
Male§¶	0	2 (2.5)	5 (5.6)
Female‖,**	4 (3.8)	10 (8.8)	8 (7.4)
Osmotic diuresis-related AEs			
Pollakiuria††	1 (0.5)	5 (2.6)	6 (3.0)
Polyuria‡‡	0	0	6 (3.0)
Volume-related AEs			
Postural dizziness	0	1 (0.5)	2 (1.0)
Orthostatic hypotension	0	0	2 (1.0)

AE, adverse event; CANA, canagliflozin; PBO, placebo; UTI, urinary tract infection.

* All AEs are reported for regardless of rescue medication except for osmotic diuresis- and volume-related AEs, which are reported for prior to initiation of rescue therapy.

† Possibly, probably or very likely related to study drug, as assessed by investigators.

‡ Death in the PBO group due to intracranial haemorrhage and brain hernia reported as serious AEs, and death in the CANA 100 mg group due to pneumonia, septic shock, acute renal failure and ischaemic hepatitis reported as serious AEs; neither death was considered by the reporting investigator to be drug-related.

§ PBO, n = 88; CANA 100 mg, n = 81; CANA 300 mg, n = 89.

¶ Including balanitis, balanitis candida, balanoposthitis and genital infection fungal.

‖ PBO, n = 104; CANA 100 mg, n = 114; CANA 300 mg, n = 108.

** Including vaginal infection, vulvitis, vulvovaginal candidiasis, vulvovaginal mycotic infection and vulvovaginitis.

†† Increased urine frequency.

‡‡ Increased urine volume.

The incidence of genital mycotic infections was higher in males and females with canagliflozin compared with placebo (Table 4); these AEs were generally mild to moderate in severity, treated with topical and/or oral antifungal therapies and resolved without interruption of study drug treatment. There was a modest increase in UTIs with canagliflozin 100 and 300 mg compared with placebo; there were no upper UTIs based on assessment of reported terms, and all events were mild to moderate in severity and none led to study discontinuation. AEs related to osmotic diuresis [i.e. pollakiuria (urine frequency), polyuria (urine volume)] and reduced intravascular volume (i.e. postural dizziness, orthostatic hypotension) were low (≤3.0% per specific AE) and led to few study discontinuations. The percentage of subjects with documented hypoglycaemia was similar with canagliflozin 100 and 300 mg and placebo (3.6, 3.0 and 2.6%, respectively), with no report of severe hypoglycaemia.

Overall, only small changes from baseline in clinical laboratory parameters were observed with canagliflozin relative to placebo at 26 weeks (Table 5). Modest improvements in indices of liver function, including alanine aminotransferase (ALT) and alkaline phosphatase, were observed with canagliflozin relative to placebo. Moderate increases in blood urea nitrogen (BUN) and slight increases in serum creatinine were seen with both canagliflozin doses compared with placebo. Serum urate was moderately decreased with both canagliflozin doses compared with placebo. Small increases in haemoglobin were observed with canagliflozin, whereas a slight decrease was observed with placebo.

**Table 5:** Mean percent changes in clinical laboratory parameters from baseline to week 26 (main study)*

	PBO	CANA 100 mg	CANA 300 mg
ALT			
Mean baseline (U/l)	26.9	27.5	28.9
Mean (s.d.) percent change	0.5 (38.3)	−11.9 (28.3)	−14.2 (30.0)
Alkaline phosphatase			
Mean baseline (U/l)	78.8	81.6	82.5
Mean (s.d.) percent change	1.7 (14.1)	0.4 (16.1)	−2.4 (15.0)
Bilirubin			
Mean baseline (µmol/l)	9.2	9.1	9.6
Mean (s.d.) percent change	5.7 (37.3)	9.8 (39.4)	2.7 (40.2)
BUN			
Mean baseline (mmol/l)	5.3	4.9	5.3
Mean (s.d.) percent change	3.1 (24.1)	20.4 (33.5)	17.7 (29.5)
Creatinine			
Mean baseline (µmol/l)	74.0	71.8	73.1
Mean (s.d.) percent change	1.9 (10.1)	2.8 (12.5)	3.5 (11.2)
Urate			
Mean baseline (µmol/l)	333.1	320.0	326.3
Mean (s.d.) percent change	1.5 (16.9)	−13.7 (17.1)	−14.6 (16.7)
Haemoglobin			
Mean baseline (g/l)	143.8	143.3	145.0
Mean (s.d.) percent change	−0.2 (6.5)	3.9 (6.0)	3.6 (5.4)

ALT, alanine aminotransferase; BUN, blood urea nitrogen; CANA, canagliflozin; PBO, placebo; s.d., standard deviation.

* Statistical comparison for CANA 100 and 300 mg versus PBO not performed (not pre-specified).

#### High Glycaemic Substudy

Canagliflozin 100 and 300 mg were generally well tolerated in the substudy, with a rate of overall AEs (61.7 and 50.0%, respectively) similar to that observed in the main study (Table S2). There were only two discontinuations due to AEs (one in each treatment group) and one serious AE (not considered related to the study drug). Rates of genital mycotic infections and UTIs were generally consistent with those observed in the main study; no AEs related to reduced intravascular volume (e.g. postural hypotension), and only one AE related to osmotic diuresis (e.g. pollakiuria), were reported. There were no reports of hypoglycaemia in the substudy. Similar to the main study, small differences in the change from baseline for serum creatinine, BUN and serum urate were observed in the two canagliflozin groups (Table S3).

## Discussion

In this study of subjects with T2DM who had inadequate glycaemic control with diet and exercise, treatment with canagliflozin 100 and 300 mg provided clinically important and statistically significant improvements in glycaemic control compared with placebo over 26 weeks; these improvements were associated with weight loss with both doses of canagliflozin. Both canagliflozin doses were generally well tolerated and were associated with a low incidence of hypoglycaemia. Of note, this study included subjects with normal renal function as well as those with mild or moderate renal impairment (chronic kidney disease, Stages 2 and 3), with an exclusion criterion of eGFR <50 ml/min/1.73 m^2^.

In the main study, reductions in HbA1c, FPG and PPG were observed with both canagliflozin doses. A large proportion of subjects reached HbA1c <7.0% with canagliflozin, and few subjects required glycaemic rescue therapy. Relative to canagliflozin 100 mg, canagliflozin 300 mg provided greater effects on glycaemic endpoints, body weight and systolic BP; similar increases in HDL-C were observed with both doses. Because canagliflozin 300 mg provides more sustained maximal decrease in RT_G_ than canagliflozin 100 mg, the incremental efficacy observed was anticipated. Doses of canagliflozin >200 mg have been reported to decrease post-meal glucose excursions, potentially through delayed glucose absorption (related to transient inhibition of the gut SGLT1 transporter due to high gut luminal concentrations of drug prior to drug absorption) [12,14,15], which could also contribute to the incrementally greater efficacy seen with canagliflozin 300 mg. Clinical mechanism of action studies [14,15] have confirmed delayed gastrointestinal glucose absorption with canagliflozin 300 mg in healthy volunteers and subjects with T2DM.

Addressing obesity is an important part of T2DM management, helping to lower insulin resistance and contributing to improvements in glycaemic control [1,16]. In addition to providing glycaemic improvements, canagliflozin treatment provided body weight reductions in this study. Because many of the traditional therapies for T2DM result in weight gain, the added benefit of weight loss with canagliflozin is clinically useful [17]. Canagliflozin was also associated with significant decreases in systolic BP (with no compensatory increase in pulse rate) and increases in HDL-C, but showed modest, dose-related increases in LDL-C. Smaller increases than those in LDL-C were observed in non–HDL-C and Apo B; the LDL-C/HDL-C ratio was slightly decreased across groups. The mechanism for the LDL-C increase with canagliflozin is not known, but may be related to the metabolic changes associated with UGE. Improvements in HDL-C and triglycerides are likely related to the improved glycaemic control and weight loss associated with canagliflozin. Taken together, multiple CV risk factors were positively modified in patients treated with canagliflozin. However, the impact of these changes on the overall risk for CV events should be further evaluated in larger CV outcome studies.

Improvements in fasting measures of βCF (HOMA2-%B, proinsulin/insulin ratio and proinsulin/C-peptide ratio) and AUC_C_/AUC_G_ ratio during the FS-MMTT were seen with canagliflozin, consistent with previous reports [11,18]. Because SGLT2 transporters are not present on β-cells, a direct mechanism for this improvement is unlikely; the improvements in βCF likely reflect reversal of glucotoxicity [19], and are possibly related to an ‘unloading’ of the β-cell as glucose is partitioned out of the system through increased UGE. In this study, sustained glucose lowering was observed through 26 weeks; because deterioration of βCF underlies disease progression, the sustained glucose lowering observed may suggest improved durability; however, longer term assessments are needed to clarify this issue.

Canagliflozin was generally well tolerated, with a safety profile consistent with expectations based on earlier reports for SGLT2 inhibitors [8,11,13,20–22]. Slightly higher rates of overall AEs were seen with canagliflozin compared with placebo, primarily due to higher incidences of UTIs and genital mycotic infections, but these led to few discontinuations. Given the increase in UGE with canagliflozin, which induces an osmotic diuresis, the increased incidence of the related AEs of polyuria and pollakiuria was as expected. However, these events were generally mild or moderate in intensity and did not lead to discontinuations. Moreover, AEs related to reduced intravascular volume were infrequent and did not lead to discontinuations. Incidences of documented hypoglycaemia with canagliflozin were low and similar to those with placebo, with no report of severe hypoglycaemia in any group; this low incidence of hypoglycaemia was expected, as the reduction in RT_G_ with canagliflozin has been reported to be in the 4.4 to 5.0 mmol/l range – above the threshold for hypoglycaemia – so that minimal further loss of urinary glucose would occur with canagliflozin as glucose levels are lowered close to the hypoglycaemic threshold [8,11].

In the high glycaemic substudy, both canagliflozin doses substantially improved glycaemic parameters and showed improvements in body weight, BP and HDL-C, similar to results from the main study. Notably, despite the markedly elevated baseline HbA1c (>10.0 and ≤12.0%) in this cohort, single-agent treatment with canagliflozin resulted in 11.6 to 17.4% of subjects reaching HbA1c <7.0%. Because UGE is proportional to the glucose concentration above RT_G_, subjects with higher baseline glucose levels might be expected to show greater osmotic diuresis and potentially increased safety and tolerability issues with canagliflozin treatment. Notably, the safety and tolerability profile of canagliflozin in the high glycaemic cohort was similar to that seen in the main study population, with minimal occurrence of AEs related to osmotic diuresis (e.g. pollakiuria) or reduced intravascular volume (e.g. postural dizziness).

In conclusion, canagliflozin 100 and 300 mg significantly improved glycaemic control, reduced body weight and were generally well tolerated compared with placebo over 26 weeks in subjects with T2DM inadequately controlled with diet and exercise, suggesting that canagliflozin may be a useful therapeutic option in this setting.

## Supporting Information

**Figure S1**. Change in HbA1c from baseline to week 26 by baseline HbA1c subgroup (LOCF). CANA, canagliflozin; HbA1c, haemoglobin A1c; LOCF, last observation carried forward; LS, least squares; PBO, placebo; s.e., standard error.

**Table S1**. Changes from baseline in efficacy endpoints at week 26 last observation carried forward (high glycaemic substudy).

**Table S2**. Summary of safety and tolerability findings (high glycaemic substudy).^*^

**Table S3**. Mean percent changes in clinical laboratory parameters from baseline to week 26 (high glycaemic substudy).
