# Supplementary material for: Efficacy and safety of canagliflozin monotherapy in subjects with type 2 diabetes mellitus inadequately controlled with diet and exercise
Source: Diabetes Obes Metab. 2013 Jan 24;15(4):372–82. doi: 10.1111/dom.12054 (PMC3593184; doi:10.1111/dom.12054)
Supplement: Supplementary file 1 [file dom0015-0372-SD1.doc]

**Appendix Figure 1.** Change in HbA_1c_ from baseline to Week 26 by baseline HbA_1c_ subgroup (LOCF).

**
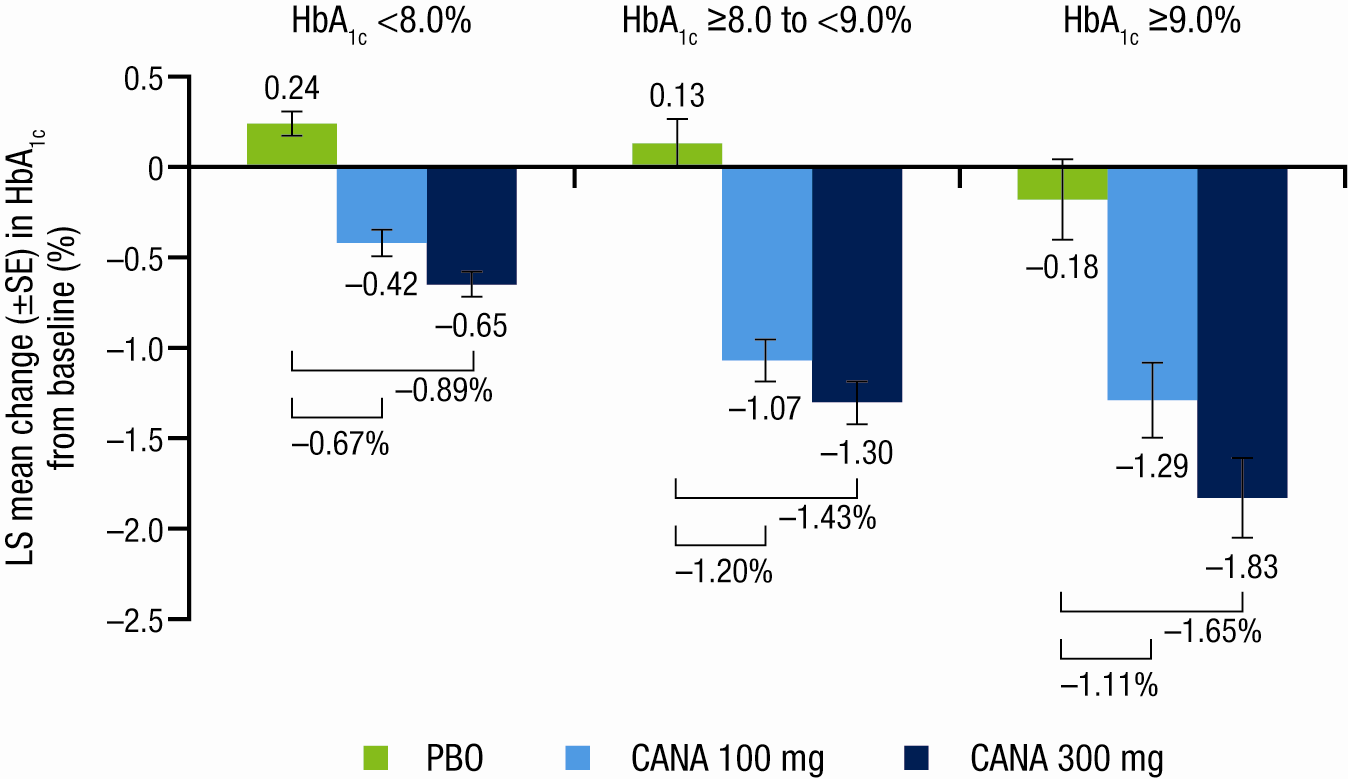
**

LOCF, last observation carried forward; LS, least squares; SE, standard error; PBO, placebo; CANA, canagliflozin.
