# Supplementary material for: Efficacy and safety of canagliflozin monotherapy in subjects with type 2 diabetes mellitus inadequately controlled with diet and exercise
Source: Diabetes Obes Metab. 2013 Jan 24;15(4):372–82. doi: 10.1111/dom.12054 (PMC3593184; doi:10.1111/dom.12054)
Supplement: Supplementary file 2 [file dom0015-0372-SD2.doc]

**Appendix Table 1. Changes from baseline in efficacy endpoints at week 26 LOCF (high glycaemic substudy)**

|  | **CANA 100 mg**  **(n = 47)** | **CANA 300 mg**  **(n = 44)** |
| --- | --- | --- |
| HbA_1c_, % |  |  |
| LS mean (SE) change | –2.1 (0.2) | –2.6 (0.2) |
| Subjects achieving HbA_1c_ <7.0% |  |  |
| n (%) | 8 (17.4) | 5 (11.6) |
| FPG, mmol/l |  |  |
| LS mean (SE) change | –4.5 (0.4) | –4.8 (0.4) |
| 2-hour PPG, mmol/l |  |  |
| LS mean (SE) change | –6.6 (0.6) | –7.0 (0.5) |
| Body weight, kg |  |  |
| LS mean (SE) percent change | –3.0 (0.6) | –3.8 (0.6) |
| Systolic BP, mmHg |  |  |
| LS mean (SE) change | –4.5 (1.8) | –5.0 (1.8) |
| Diastolic BP, mmHg |  |  |
| LS mean (SE) change | –3.5 (1.2) | –2.2 (1.2) |
| Triglycerides, mmol/l |  |  |
| LS mean (SE) percent change | –0.6 (7.4) | –12.8 (7.5) |
| HDL-C, mmol/l |  |  |
| LS mean (SE) percent change | 2.3 (2.9) | 10.7 (2.9) |
| LDL-C, mmol/l |  |  |
| LS mean (SE) percent change | –5.9 (4.4) | 2.9 (4.4) |

LOCF, last observation carried forward; CANA, canagliflozin; LS, least squares; SE, standard error; FPG, fasting plasma glucose; PPG, postprandial glucose; BP, blood pressure; HDL-C, high-density lipoprotein cholesterol; LDL-C, low-density lipoprotein cholesterol.
