# Supplementary material for: Efficacy and safety of canagliflozin monotherapy in subjects with type 2 diabetes mellitus inadequately controlled with diet and exercise
Source: Diabetes Obes Metab. 2013 Jan 24;15(4):372–82. doi: 10.1111/dom.12054 (PMC3593184; doi:10.1111/dom.12054)
Supplement: Supplementary file 3 [file dom0015-0372-SD3.doc]

**Appendix Table 2. Summary of safety and tolerability findings (high glycaemic substudy)***

|  | **Subjects, n (%)** | |
| --- | --- | --- |
|  | **CANA 100 mg**  **(n = 47)** | **CANA 300 mg**  **(n = 44)** |
| Any AE | 29 (61.7) | 22 (50.0) |
| AEs leading to discontinuation | 1 (2.1) | 1 (2.3) |
| AEs related to study drug^†^ | 8 (17.0) | 5 (11.4) |
| Serious AEs | 0 | 1 (2.3) |
| Deaths | 0 | 0 |
| **Selected AEs** |  |  |
| UTI | 3 (6.4) | 2 (4.5) |
| Genital mycotic infection |  |  |
| Male^‡,§^ | 1 (4.3) | 1 (5.3) |
| Female|,¶ | 5 (20.8) | 1 (4.0) |
| Osmotic diuresis-related AEs |  |  |
| Pollakiuria^#^ | 0 | 1 (2.3) |

CANA, canagliflozin; AE, adverse event; UTI, urinary tract infection.

^*^All AEs are reported regardless of rescue medication, except for osmotic diuresis-related AEs, which are reported prior to initiation of rescue therapy.

^†^Possibly, probably, or very likely related to study drug, as assessed by investigators.

^‡^CANA 100 mg, n = 23; CANA 300 mg, n = 19.

^§^Including balanitis.

^|^CANA 100 mg, n = 24; CANA 300 mg, n = 25.

^¶^Including vaginal infection, vulvovaginal candidiasis, vulvovaginal mycotic infection, and vulvovaginitis.

^#^Increased urine frequency.
