# Supplementary material for: Efficacy and safety of canagliflozin monotherapy in subjects with type 2 diabetes mellitus inadequately controlled with diet and exercise
Source: Diabetes Obes Metab. 2013 Jan 24;15(4):372–82. doi: 10.1111/dom.12054 (PMC3593184; doi:10.1111/dom.12054)
Supplement: Supplementary file 4 [file dom0015-0372-SD4.doc]

**Appendix Table 3. Mean percent changes in clinical laboratory parameters from baseline to week 26 (high glycaemic substudy)**

|  | **CANA 100 mg** | **CANA 300 mg** |
| --- | --- | --- |
| ALT |  |  |
| Mean baseline, U/l | 28.5 | 31.3 |
| Mean (SD) percent change | –9.2 (54.1) | 23.9 (226.0) |
| Alkaline phosphatase |  |  |
| Mean baseline, U/l | 101.9 | 98.1 |
| Mean (SD) percent change | –9.5 (9.7) | –6.3 (17.7) |
| Bilirubin |  |  |
| Mean baseline, μmol/l | 8.8 | 10.0 |
| Mean (SD) percent change | 5.5 (31.0) | –2.2 (32.6) |
| BUN |  |  |
| Mean baseline, mmol/l | 4.7 | 4.8 |
| Mean (SD) percent change | 21.2 (29.8) | 27.3 (32.9) |
| Creatinine |  |  |
| Mean baseline, μmol/l | 72.1 | 68.7 |
| Mean (SD) percent change | 0.4 (11.9) | 3.1 (11.9) |
| Urate |  |  |
| Mean baseline, μmol/l | 286.2 | 285.6 |
| Mean (SD) percent change | –0.3 (19.3) | –1.4 (17.3) |
| Haemoglobin |  |  |
| Mean baseline, g/l | 147.3 | 151.8 |
| Mean (SD) percent change | 2.5 (6.3) | 1.3 (6.2) |

CANA, canagliflozin; ALT, alanine aminotransferase; SD, standard deviation; BUN, blood urea nitrogen.
